# Precise Analysis of Polymer Rotational Dynamics

**DOI:** 10.1038/srep19127

**Published:** 2016-01-08

**Authors:** Jun Mo Kim, Chunggi Baig

**Affiliations:** 1 School of Energy and Chemical Engineering, Ulsan National Institute of Science and Technology (UNIST), Ulsan 689-798, South Korea

## Abstract

Through the analysis of individual chain dynamics alongside the corresponding molecular structures under shear via nonequilibrium molecular dynamics simulations of C_178_H_358_ linear and short-chain branched polyethylene melts under shear flow, we observed that the conventional method based on the chain end-to-end vector (and/or the gyration tensor of chain) is susceptible to quantitatively inaccurate measurements and often misleading information in describing the rotational dynamics of polymers. Identifying the flaw as attributed to strong irregular Brownian fluctuations inherent to the chain ends associated with their large free volume and strong molecular collisions, we propose a simple, robust way based on the chain center-to-center vector connecting the two centers of mass of the bisected chain, which is shown to adequately describe polymer rotational dynamics without such shortcomings. We present further consideration that the proposed method can be useful in accurately measuring the overall chain structure and dynamics of polymeric materials with various molecular architectures, including branched and ring polymers.

Among a wide spectrum of characteristic time scales intrinsic to polymers, the time scale involving whole chain rotational motion (generally considered the longest time scale) is perhaps the most important as it is closely related with large-scale chain structure and dynamics^1^,^2^,^3^. A precise knowledge of chain rotational dynamics is essential for understanding the rheological properties and phenomena of polymeric materials undergoing shear flow. As such, considerable research efforts have been made in revealing the microscopic details of rotational dynamics of polymers during the past decades using advanced experiment and simulation techniques^4^,^5^,^6^,^7^. The conventional method for measuring the rotational dynamics of polymer chains is based on the chain end-to-end vector **R**_ete_ that, representing the largest length scale of a polymer, has been widely applied to evaluate the overall chain orientation and dynamical correlation functions^8^,^9^,^10^,^11^,^12^,^13^,^14^. However, due to their large surrounding free volume and associated high degree of molecular collisions with other chains under flow, the outermost chain-end atoms display a strong and fast irregular motion compared with the inner atoms of the chain. This fast random movement conveyed at the chain ends can give rise to inaccurate or false information regarding chain dynamics, including rotation and tumbling in shear. We therefore aim to clarify this through a detailed analysis of individual chain dynamics alongside the corresponding molecular structures under shear, via direct nonequilibrium molecular dynamics (NEMD) simulations. In this work, we have comprehensively explored the possible erroneous information that can occur in polymer systems by tracking individual chain motions over the course of the full rotational time scale. Our results show that the conventional method, based on the chain end-to-end vector, can often produce quantitatively inaccurate measurements or misleading information on chain dynamics associated with rotation and tumbling mechanisms, especially under an external flow field. This may eventually lead to imprecise results with regards to the structural and dynamical properties of polymeric systems, including the rotational time spectrum, viscosity, and orientation angle distributions.

Taking into account the origins of such properties, we herein propose a simple but robust way to correctly describe the rotational dynamics of polymers. This new method utilizes the chain center-to-center vector, **R**_ctc_, connecting the two centers of mass formed by dividing the chain into two equal fragments. It will be shown here that the **R**_ctc_ vector adequately describes the actual chain dynamics by effectively removing the flaws that could be produced by the **R**_ete_. In addition, we show that dynamical analysis based on **R**_ctc_ provides useful information about the overall molecular shape and structure, which, together with rotational dynamics information, is essential to aid the understanding of the stress relaxation behavior of various kinds of linear, branched, and ring polymeric materials.

Linear and short-chain branched (SCB, with each chain containing 128 carbon atoms in the backbone and 5 carbon atoms on each of its 10 branches) polyethylene melts of the same molecular formula C_178_H_358_, were investigated using atomistic Canonical (*NVT*) NEMD simulations for planar Couette flow at constant temperature *T* = 450 K and density *ρ* = 0.7818 g/cm^3^, implemented with the Nosé-Hoover thermostat^15^,^16^,^17^ and the Lees-Edwards sliding-brick boundary conditions^18^,^19^. The TraPPE (Transferrable Potentials for Phase Equilibria) united-atom potential model^20^ was employed in the simulations, and the set of the evolution equations was numerically integrated using an efficiently constructed *r*-RESPA (reversible Reference System Propagator Algorithm)^21^ with two different time scales for an MD step: 2.39 fs for the two non-bonded [inter- and intra-molecular Lennard-Jones (LJ)] interactions, and 0.48 fs for the three bonded (bond-stretching, bond-bending, and bond-torsional) interactions (see ref. 22 for more details of simulations). For each system, we employed a sufficiently large (considering stretched chain dimension) rectangular simulation box enlarged in the flow direction to avoid system-size effects. This was particularly important at high shear rates where molecules become highly stretched and aligned: i.e., 216 chains were present in a volume of 264 × 66 × 66 Å^3^ (*x* × *y* × *z*) for the linear polymer, and 162 chains in 198 × 66 × 66 Å^3^ for the SCB polymer, with the *x-* and *y*-axes being the flow and the velocity gradient directions, respectively. The systems were subjected to a broad range of flow rates of 0.39 ≤ *Wi* ≤ 7000, where the Weissenberg number (*Wi*) is defined as the product of the longest relaxation time (*λ*) of the system and the imposed shear rate. At present, we estimated *λ* for each system in the conventional way based on the time autocorrelation function of the unit chain end-to-end vector by evaluating the integral below the typical stretched-exponential curve describing the function; the result is that *λ* = 15.6±1.0 ns for the linear, and *λ* = 21.7±1.5 ns for the SCB polymer, which basically corresponds to the longest (the first mode) relaxation time of the Rouse theory.

We first examine the rotational motion exhibited by an individual chain under shear flow, as represented by the chain orientation angle *θ* with respect to the flow direction [here we chose the range of *θ* = 0°~360° (rather than *θ* = −90°~90° or *θ* = 0°~180°) to see one full cycle of the chain rotation in a continuous manner]. Figure 1 shows a temporal variation of two different types of *θ* for a randomly selected SCB chain, with one being computed based on the chain end-to-end vector **R**_ete_, and the other based on the chain center-to-center vector **R**_ctc_ (connecting the two centers of mass of the bisected chain). Figure 1a displays a typical tumbling cycle of a polymer chain. It should be noted that the majority of chain rotation during tumbling cycles occurs in a highly ellipsoidal, hairpin shape, with the two orientation angles shown in the figure representing a typical semi-periodic tumbling cycle of chain molecules. However, overall, the *θ*-values obtained from the **R**_ete_ appear to fluctuate much more frequently and with a larger magnitude compared to those from the **R**_ctc_. In particular, in the range of *t* = 0.07−0.09 ns, the *θ*-values obtained from the **R**_ete_ were found to fluctuate rapidly around *θ* = 0° or 360°, giving rise to the impression of a chain wagging motion, although the entire chain body was found to rotate in a relatively continuous manner during the cycle. This rather artificial chain motion is ascribed to the fast, random Brownian fluctuation inherent to the chain ends, which can move freely without much influence of the flow fields. However, such misleading chain dynamics do not show up in the case of **R**_ctc_, with a smooth rotation behavior being correctly captured. A clearer case of such motion being produced by the **R**_ete_ can be seen in the *θ* data between *t* = 0.12 ns and 0.20 ns. In this time range, the chain exhibits a wagging motion between *t* = 0.12 ns and 0.18 ns, followed by an overall chain rotation between *t* = 0.19 ns and 0.20 ns. Furthermore, this wagging motion occurs three times between *θ* = 100° and 220° during the time span. This behavior is correctly described by the *θ*-values of **R**_ctc_. In contrast, the *θ*-values of **R**_ete_ display a number of crossovers during the same time range. In addition, the range of wagging angle exhibited by the **R**_ete_ appears to be much larger (20° ≤ *θ* ≤ 310°) than the true one. Again, this discrepancy is supposed to originate from the random Brownian fluctuation of the chain ends. Figure 1b depicts another example where it can be seen that the **R**_ete_ result exhibits two seemingly (spurious) tumbling motions at approximately *t* = 0.76 ns and 0.82 ns, although the whole chain actually fluctuates in a narrow range (−5° ≤ *θ *≤ 5°) with respect to the flow direction. The real tumbling event occurs at approximately *t* = 0.82 ns, which is precisely captured by the **R**_ctc_. In addition, it is noticed that the *θ*-values of **R**_ete_ apparently display the tumbling event as being lengthened through *t* = 0.80 ns and 0.88 ns. In Fig. 1c, we directly illustrate via a series of instantaneous snapshots of an individual chain motion over a short time, how the two vectors described by **R**_ete_ and **R**_ctc_ can quantitatively be very different from each other, and which of the two represents the true chain conformation more accurately. As correctly interpreted by the **R**_ctc_, the whole chain conformation and its spatial orientation does not vary significantly, although the **R**_ete_ erroneously shows a large variation in the chain orientation. These results confirm that the principal chain orientation, the overall chain structure, and the detailed chain dynamics are adequately described by the **R**_ctc_.

In an effort to determine the most suitable method for enabling a precise description of the rotational dynamics of chain molecules, we carried out a systematic procedure involving varying the descriptive connection vector **R** based on the Kuhn length scale. This was carried out taking into account the above results. Furthermore, in order to analyze the connection between a large-scale chain structure and **R**, we performed an eigenvector analysis on the gyration tensor of chain **G** (*G*_αβ_, α,β = *x*,*y*,*z*; 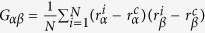 and 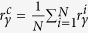 with **r**^*i*^ being the position vector of the *i*^th^ atom of a chain composed of *N* atoms) whose principal diagonal components represent the overall chain dimension in a three-dimensional space. Figure 2a shows an example of a time-dependent motion of a selected C_178_H_358_ linear chain where several different orientation angles were obtained via different methods (e.g., **R**_ete_, **R**_ctc_, and the director vector of **G**). First, it is seen that the variation of the largest eigenvalue of **G** correlates well with the chain dynamics, i.e., when a chain is stably aligned to the flow direction, the eigenvalue reaches a maximum, but when it transitions into tumbling, the eigenvalue drops rapidly to reach a minimum value. Based on this observation, it might be expected that the orientation angle based on the director vector could represent the overall chain motion. However, as clearly shown in Fig. 2a, the *θ*-values from the director do not accurately describe the overall chain orientation during rotation, as they appear to show a continuous wagging motion of the chain around the flow direction. This behavior is specifically depicted in Fig. 2b. It is seen that although the entire chain performs a typical tumbling movement, the director vector can only fluctuate around the flowing direction, thus resulting in a false wagging motion. Therefore, while the gyration tensor can represent the overall conformational changes of the polymer chain, it is generally not able to adequately describe the rotational dynamics of individual chains.

We now analyze in detail the results for the *θ*-values obtained from **R**_ete_ and **R**_ctc_. Besides large statistical fluctuations, the *θ*-values of **R**_ete_ is found to exhibit a quantitatively delayed chain tumbling mechanism at *t* = 0.17 ns and 0.39 ns compared to those of **R**_ctc_. Furthermore, as evident from the snapshots of random chain conformations taken between *t* = 0.371 ns and 0.377 ns (Fig. 2c), the *θ*-values of the **R**_ctc_ (from 144° to 93° to 41°) correctly describe the overall chain rotational motion, whereas the **R**_ete_ displays a small degree of chain rotation (exhibiting an unstable transition) close to 180°. Interestingly, the maximum, minimum, and transient behaviors represented by the eigenvalue of **G**, are remarkably well matched to the *θ*-values calculated from the **R**_ctc_.

Based on these results, we devised alternative representations of **R** based on the Kuhn length scale (equivalent to approximately 11 carbon atoms in the present polyethylene melts) to study the consequences of systematically reducing the effect of random Brownian motions of the chain ends. An individual linear chain was divided into 4, 8, and 16 segments (denoted by **R**_Kuhn(4)_, **R**_Kuhn(8)_, and **R**_Kuhn(16)_, respectively) and a vector constructed connecting the two outermost parts. It is noted that in the case of **R**_Kuhn(16)_, one segment is approximately equal to one Kuhn segment. As can be seen in Fig. 2a, the orientation angles calculated from the **R**_Kuhn(8)_ are located between those from the **R**_ete_ and the **R**_ctc_. It is evident that with a decrease in the Kuhn number (16→8→4), the calculated *θ*-values are closer to those of **R**_ctc_, as also confirmed in this study (data not shown here). This phenomenon is physically conceivable, as the Brownian effect of the chain ends becomes smaller with an increase in end segment size. **R**_ctc_ is considered a limiting case of such a chain-size reduction procedure, representing a connecting vector of the largest possible segments. It can therefore be concluded from these results that the **R**_ctc_ vector is a promising quantity for the description of polymer chain dynamics. In addition, it is further considered to be one of the best measures for representing the chain structure or conformation, as it is correlates well with the overall trends of the eigenvalue of **G**.

Despite such discrepancies between different representations of individual chain dynamics, average physical quantities may not display such differences due to cancelling effects. We therefore chose to investigate further the average values of important physical quantities, such as the probability distribution function (PDF) of the chain orientation angle, and the time correlation function 〈**u**(*t*)·**u**(*t* + *τ*)〉 based on the chain end-to-end segments. Four spatial orientation regions were selected, as depicted in Fig. 3a. As seen in the plot in Fig. 3a, as *Wi* increases, molecules become more aligned with the flow direction, with the PDFs of Regions 1 and 4 increasing, and those of Regions 2 and 3 decreasing. Although qualitatively similar, there appear to be quantitatively distinct results between the **R**_ete_ and the **R**_ctc_. Most importantly, the PDF of the favorable Region 1 appears to have smaller values in the **R**_ete_ compared to the **R**_ctc_ in the full range of *Wi* numbers studied, with the discrepancy being largest at an intermediate *Wi* number. Correspondingly, the PDFs of the other regions is seen to be higher in the **R**_ete_ compared to the **R**_ctc_. Indeed, the **R**_ete_ is found to underestimate the residence time of the most favorable chain orientation. This is again attributed to a fast random Brownian motion from the chain ends. Figure 3b displays the result of the time correlation function 〈**u**(*t*)·**u**(*t* + *τ*)〉 at both a low and a high *Wi* number. It can be seen from these results that the **R**_ete_ exhibits a significantly faster decay of the time correlation function compared to the **R**_ctc_ (the relaxation times computed using a stretched exponential function are found to be 17.96 ns and 23.32 ns at *Wi* = 1.3 for the **R**_ete_ and the **R**_ctc_, respectively). Once again, this behavior is expected to be due to the Brownian fluctuation of the chain ends, which is particularly effective at relatively weak flow regimes. In addition, it is seen that the result of the **R**_Kuhn_ having a smaller Kuhn number exhibits a less discrepancy from that of the **R**_ctc_. Furthermore, the overall discrepancies appear to reduce with increasing *Wi* number, as the effect of the flow field becomes dominant over the Brownian effect of the chain ends at higher flow strength. This can be seen in the inset of Fig. 3b. Overall, the quantitative difference between **R**_ete_ and **R**_ctc_ appears to decrease with an increase in flow strength. However, we should keep in mind that similar results on average for a physical quantity measured by two different methods do not generally represent the same for all the details, e.g., the degree of statistical fluctuations and instantaneous individual chain dynamics. We should further note here that although the **R**_ete_ vector may be subject to certain ambiguities in quantifying the overall chain rotational dynamics, it is still very useful physical measure in evaluating many structural and rheological properties, such as the large-scale chain dimension and the Rouse relaxation time (which generally includes the effect of the Brownian motion of the chain ends as well, and the correlation time based on the **R**_ctc_ reflects the pure overall rotational dynamics of chains).

We now put forward consideration of several cases where the proposed method based on **R**_ctc_ can be useful in measuring the overall chain structure and dynamics of polymeric materials. In Fig. 4, we assess their adequacy of **R**_ete_, **G**, and **R**_ctc_ in describing the actual chain dynamics accompanying structural changes that can occur in various types of polymers at equilibrium or nonequilibrium states. Figure 4a depicts a case where a linear chain exhibits a transition of its overall shape weighted from the left to the right. It is seen that while the **R**_ctc_ vector is able to properly capture the transient chain conformation, the **R**_ete_ is kept constant staying at its original position without reflecting any of the structural change. Figure 4b presents another situation that can occur in the case of an H-shaped branched polymer, where branches 1 and 2 are supposed to interchange their positions by rotation around the main backbone. If we choose the **R**_ete_ as connecting between the ends of branches 1 and 4 (based on the longest chain dimension), the **R**_ete_ may seem to indicate a wagging or tumbling motion of the chain, which, however, has not actually performed any of the overall rotation, as well described by the **R**_ctc_. Another situation worthy of consideration is illustrated in Fig. 4c where a ring chain displays a structural deformation and a tank-treading rotational dynamics (as one of the major dynamical mechanisms specific to ring polymers^23^). Due to the lack of chain ends, ring chains are very often likely to carry out a structural deformation without overall chain rotation. It is seen in Fig. 4c that a pure (e.g., compressive) deformation in ring structure without rotation is adequately represented by the **R**_ctc_ whereas the director of the gyration tensor **G** seems to erroneously convey misleading information, i.e., a pure rotation of the chain without structural deformation. As regards a tank-treading rotational motion of ring chain, it is seen that while the **R**_ctc_ can correctly describe the tank-treading motion of chain, the **G** would not be able to provide the corresponding information. In addition, for a dynamical mechanism where some extruded chain segments (excursions) diffuse along a ring chain (particularly important under shear flow), the **R**_ctc_ can still provide an adequate representation, but the ring diameter vector **R**_d_ (similarly to **R**_ete_ in linear polymer) is seen to be incapable of the description. Figure 4d illustrates a structural change displayed by a star-shaped chain with arm fluctuations (e.g., flipping, rotation, etc.) but without overall chain rotation; here again the **R**_ctc_ is seen to properly represent the structural behavior whereas the **G** would erroneously inform the whole chain rotation by 90°. [There are several different, but equally useful, ways of choosing the **R**_ctc_ vector to describe the chain dynamics of star polymers. It would be possible to devise the various potentially useful **R**_ctc_ vectors and combine information from each representation to give details of both the local and global chain structure and dynamics.]

These ideas indicate that the **R**_ctc_ vector can be a useful measure to describe not only the chain dynamics but also the overall shape and structure of polymer chains with various molecular architectures. The **R**_ctc_ vector might even be useful in estimating other rheological properties or behavior of polymer systems such as the mechanism for stress relaxation. In addition, extending the proposed method by properly adjusting the number and the location of Kuhn segments along the chain and applying the local **R**_ctc_ vector for each segment, we may be able to obtain more detailed structural and dynamical information of polymers with complicated molecular architectures such as hyperbranched polymers and dendrimers. For example, in the case of an asymmetrical branched polymer system, we can divide the whole molecule into the main backbone and each of the branches and then set the local **R**_ctc_ vector for the individual part. However, even in this case, the global **R**_ctc_ bisecting the entire chain based on the chain center of mass would still convey useful information about the overall chain dynamics.

In this study, we have clarified some possible flaws that can often occur in the description of individual chain dynamics using conventional methods based on the chain end-to-end vector **R**_ete_. These were found to be directly associated with strong irregular Brownian fluctuations inherent to chain ends due to their large free volume and strong intermolecular collisions. We therefore devised a simple, robust method for the proper description of the overall, actual chain dynamics, based on the chain center-to-center vector **R**_ctc_ that connects the two centers of mass of the bisected chain. The results obtained using the **R**_ctc_ vector were seen to effectively remove such flaws arising from the **R**_ete_, demonstrating that **R**_ctc_ is to be an adequate quantity not only in correctly describing the dynamics of polymer chains but also in measuring the overall chain structure. We further showed that the **R**_ctc_ representation would be equally useful in describing the dynamics and structure of various branched and ring polymers. The **R**_ctc_ representation can apply equally well to other flow fields such as elongational flow or mixed shear and elongation. Since the **R**_ctc_ representation is capable of conveying relevant information about the overall shape and structural change of polymer, it would be also useful in exploring transient behavior of flowing polymeric system and not only steady-state flow. Further, it could also be beneficial in constitutive modeling. We expect that the proposed method can be widely applied in the study of polymeric fluid dynamics.

## Additional Information

**How to cite this article**: Mo Kim, J. and Baig, C. Precise Analysis of Polymer Rotational Dynamics. *Sci. Rep.* **6**, 19127; doi: 10.1038/srep19127 (2016).

## Figures and Tables

**Figure 1:**
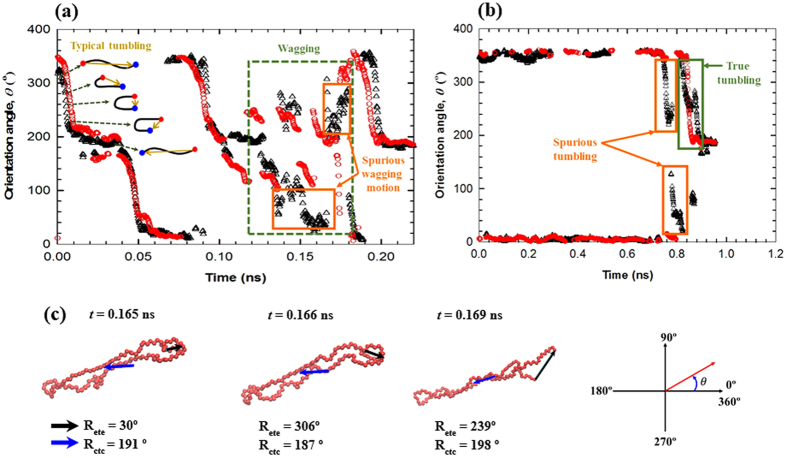
Temporal variation of chain orientation angle under shear. (**a**) Plots of the chain orientation angle *θ* with respect to the flow direction as a function of time, for a randomly selected short-chain branched polymer molecule at *Wi* = 5000. The black triangles and red circles represent the *θ*-values computed from the chain end-to-end vector **R**_ete_ and the chain center-to-center vector **R**_ctc_, respectively. A typical tumbling motion of a chain molecule under shear is schematically depicted: the yellow arrows connecting the two chain ends represent the chain end-to-end vector. The green box (broken line) indicates a time period of a chain wagging motion, and the orange boxes (solid line) indicate spurious wagging motions as predicted by the **R**_ete_. (**b**) Similar to (a) but for a tumbling motion of a linear chain at *Wi* = 5000. (**c**) Snapshots for a randomly selected SCB backbone with two types of orientation angle. The **R**_ete_ and the **R**_ctc_ vector are represented by a black and a blue arrow, respectively.

**Figure 2:**
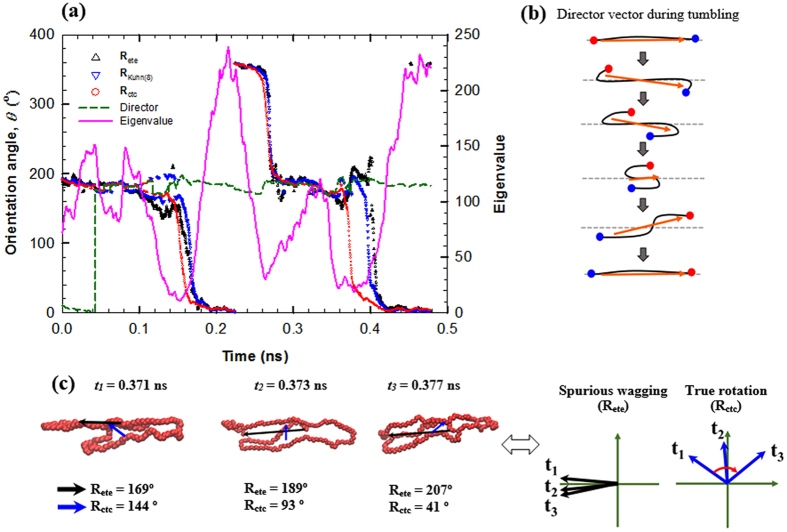
Comparison between different methods for describing chain rotational dynamics. (**a**) Plots of the chain orientation angle *θ* with respect to the flow direction as a function of time for a randomly selected linear chain using various methods at *Wi* = 5000. The largest eigenvalue of the gyration tensor and the corresponding vector (director) are also shown for comparison. (**b**) Pictorial illustration of the evolution behavior of the director during a tumbling cycle. Chain ends are represented by red and blue dots, while an orange arrow represents the director vector. (**c**) A series of instantaneous snapshots of the chain between *t* = 0.371 ns and 0.377 ns. The black and blue arrows indicate the chain end-to-end vector **R**_ete_ and the chain center-to-center vector **R**_ctc_, respectively.

**Figure 3:**
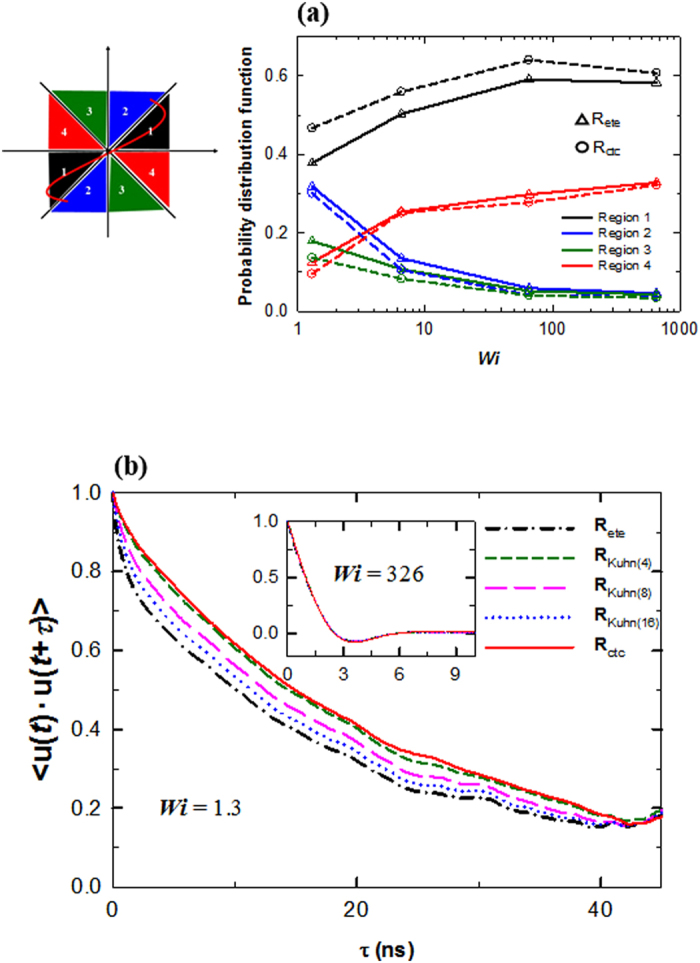
Comparison between different methods for the orientation angle distribution and time correlation function. (**a**) Probability distribution function (PDF) for the orientation angle as a function of flow strength with its classification into four different regions. (**b**) Time correlation function of the unit vector computed from a range of methods at a low *Wi* value (*Wi* = 1.3). The inset shows the result at a high *Wi* value (*Wi* = 326).

**Figure 4:**
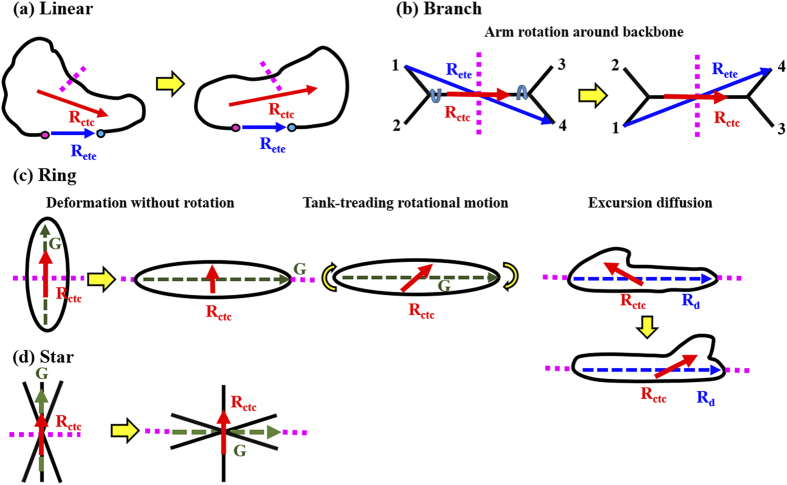
Schematic description of individual methods for rotational dynamics of polymers with various molecular architectures. Comparison of the chain end-to-end vector R_ete_, the gyration tensor G of chain, and the chain center-to-center vector R_ctc_ for their capabilities in describing the actual chain dynamics along with structural changes that can occur in various types of polymeric materials under equilibrium or flowing conditions: (**a**) Linear polymer, (**b**) Branch polymer, (**c**) Ring polymer, and (**d**) Star polymer. The bisection boundary of chain is indicated by the broken lines.
